# Opioids in the Brazilian Healthcare Landscape: Crucial Analysis through Anvisa VigiMed Data and Pharmacogenetic Aspects

**DOI:** 10.1021/acsomega.5c01527

**Published:** 2025-05-21

**Authors:** Lariana Almeida Szczesny, Raíssa Nunes dos Santos, Juliano de Oliveira Silveira, Silvana de Almeida, Júlia Pasqualini Genro, Marilu Fiegenbaum

**Affiliations:** † Programa de Pós-graduação em Biociências, 117303 Universidade Federal de Ciências da Saúde de Porto Alegre - UFCSPA, Rio Grande do Sul, Porto Alegre 90050-170, Brazil; ‡ Departamento de Ciências Básicas da Saúde, Universidade Federal de Ciências da Saúde de Porto Alegre - UFCSPA, Rio Grande do Sul, Porto Alegre 90050-170, Brazil

## Abstract

Adverse Drug Reactions (ADRs) present a significant challenge to healthcare systems, contributing substantially to hospital admissions. Opioid analgesics are widely used in the pharmacological treatment of various types of pain; however, ADRs represent a major limitation to their use. This study aims to investigate the profile of ADRs reported in Vigimed (the official Brazilian ADR reporting system) following the implementation of active surveillance through pharmacovigilance trackers. Additionally, it evaluates pharmacogenetic evidence to identify genes potentially involved in opioid-related ADRs. This retrospective cross-sectional study analyzed ADR cases reported to Vigimed from January 2018 to April 2023. Data were extracted from Vigimed, tabulated, and subjected to statistical analysis, using the reporting odds ratio (ROR) to assess the strength of associations between opioids and ADRs. During the study period, there were 238,363 ADR reports, of which 6,001 were related to opioid treatment. The distribution among opioids was as follows: 36.7% morphine, 32.4% tramadol, 21.6% fentanyl, 5.4% methadone, 3.7% codeine, and 0.2% oxycodone. The most frequent adverse events associated with opioids were cardiac disorders (ROR 1.70), skin and subcutaneous tissue disorders (ROR 2.18), gastrointestinal disorders (ROR 2.88), and nervous system disorders (ROR 1.19). Furthermore, pharmacogenetic evidence indicates that the *CYP2D6* gene has a Level 1 association for codeine and tramadol and a Level 2 association for oxycodone. These findings highlight significant associations between specific opioids and ADRs, emphasizing the influence of genetic variations on adverse reactions and the need for personalized medicine in pain management.

## Graphical abstract

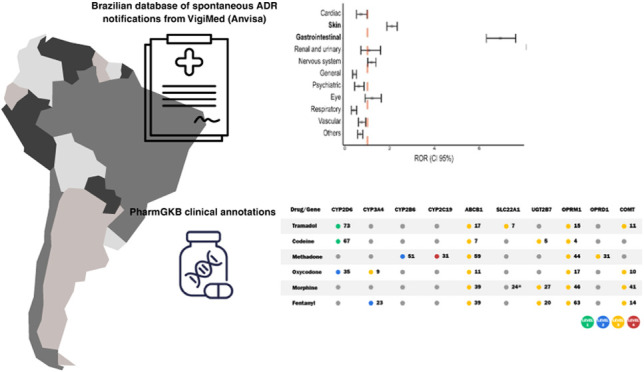

## 1 Introduction

Adverse drug reactions (ADRs) present a significant challenge in modern healthcare, driven by therapeutic complexity, an aging population, lifestyle factors, and genetic variations.^1^ In Brazil, data from the Brazilian Health Regulatory Agency (Anvisa) indicate a consistent increase in ADR reports involving both opioid and nonopioid medications, with an average of 43.7 notifications per month.^2^ Studies suggest that 5–10% of patients may experience an ADR during hospital admission, stay, or discharge, despite numerous preventive measures.^3^ The frequency of these adverse events demands attention due to their associated morbidity and mortality, financial burden, and potential to strain the prescriber-patient relationship.^1^

In pain management, pharmacological treatment should be individualized, involving a discussion of treatment expectations and a clear plan for medication duration. Chronic noncancer pain (CNCP) is highly prevalent, affecting approximately 20% of European adults.^4^ Since the 1990s, there has been a significant increase in opioid use, driven partly by efforts to improve pain management and the widespread adoption of the World Health Organization (WHO) three-stepladder for cancer pain, which has also been applied to CNCP.^5^

In the United States, opioid consumption tripled between 1999 and 2015, rising from 180 to 640 morphine milligram equivalents per capita.^6^ In Latin America, tramadol is widely used for treating severe acute pain, such as postoperative, post-traumatic, and low back pain, as well as moderate to severe chronic pain, including neuropathy, osteoarthritis, cancer pain, and fibromyalgia. It is also used for mild pain in the elderly and in patients ineligible for nonsteroidal anti-inflammatory drugs (NSAIDs). However, contrary to recommendations, opioids are often used as a standalone treatment rather than as part of a multimodal approach to pain management in these countries.^7,8^

Pharmacovigilance programs focus on ensuring the safe and rational use of approved medications by continuously monitoring their risk-benefit profiles.. Voluntary reporting of adverse events is a cornerstone of pharmacovigilance, generating the majority of data used to establish causal links between drugs and adverse events.^9,10^ Digital health records have become increasingly essential in healthcare and medical research, enhancing decision-making, diagnostics, therapeutic choices, and overall patient management. These records offer a comprehensive repository of patient information, allowing healthcare professionals to access accurate, up-to-date data efficiently. By consolidating medical histories, test results, treatments, and other relevant data into a digital format, electronic health records facilitate seamless communication among healthcare providers, enabling more coordinated and personalized patient care. Additionally, digitized health records enhance data analytics, driving research and innovation, particularly in fields such as movement disorders. In this context, VigiMed, implemented by Anvisa, became Brazil’s official adverse drug reaction (ADR) reporting system, replacing the VigiFlow platform in 2019. This digital system supports passive surveillance of suspected ADRs by collecting and analyzing individual case safety reports (ICSRs) submitted through spontaneous reporting.

Recent advances in pharmacogenomics highlight the role of genetic variants in predicting adverse effects, including the use of biological markers within pharmacovigilance.^11,12^ Genetic testing has become more accessible and is increasingly supported by strong clinical evidence. Genetic variations affect enzyme activity, drug metabolism, interactions with cellular receptors, and intracellular signaling pathways. Guidelines from organizations like the Clinical Pharmacogenetics Implementation Consortium (CPIC) and resources such as PharmGKB support genotype-based prescribing, while regulatory bodies like the FDA recommend genetic testing before initiating certain medications. Further research across diverse populations is essential to validate these findings across different ethnic and geographic groups.^13–16^

Therefore, this study aims to analyze spontaneous ADR notifications related to opioids in electronic medical records reported to Anvisa and assess pharmacogenomic evidence levels based on clinical annotations describing gene-drug interactions.

## 2 Results and Discussion

Opioid analgesics are widely utilized in the pharmacological treatment of various types of pain, both acute and chronic. Approximately 90% of patients suffering from chronic pain rely on some form of opioid. However, ADRs present significant limitations to their use.^17,18,8^ Adverse drug reactions are a significant contributor to hospital admissions and healthcare utilization, with complications estimated to affect nearly 22% of patients during their hospital stays. Between 2009 and 2018, Brazil’s Unified Health System (SUS) recorded 607,633 hospitalizations related to adverse medication reactions. Additionally, ADRs impose substantial costs on health systems, and individuals experiencing these reactions face an increased risk of mortality.^19–21^ Consequently, electronic health data is invaluable in clinical studies for the pharmacological monitoring of patients. Additionally, spontaneous notifications from the medical team serve as an economical and effective method for tracking ADRs. This study is the first to utilize the electronic notification system (VigiMed) to analyze reports of ADRs related to opioid medications in the Brazilian population between January 2018 and April 2023, VigiMed recorded 6,001 ADR reports linked to opioid treatment.

In this study, between January 2018 and April 2023, the VigiMed database recorded 155,401 ADR notifications, including 30,785 related to vaccines. After excluding vaccine-related notifications, 124,616 ADR notifications remained in the database. Among these, 238,363 ADR reports were included, with 6,001 linked to opioid treatment (36.7% morphine, 32.4% tramadol, 21.6% fentanyl, 5.4% methadone, 3.7% codeine, and 0.2% oxycodone). Women accounted for the majority of individuals affected by opioid-related ADRs (2,744 individuals, 56.42%). The highest percentage of ADRs was observed in individuals aged 18–44 years (31.97%), followed by those over 65 years (24.03%) and those aged 45–64 years (21.32%). Most notifications were made in healthcare settings (75.68%), with pharmacists accounting for 68.98% of the reports. Hospitalization occurred in 5.12% of the cases, although the majority of data (approximately 75%) did not report hospitalization (Table 1).

**Table 1:** Characteristics of Data Entry in Pharmacovigilance System (VigiMed)

	all	opioids
*n* of notifications	124,616	4864
*n* of ADR reports	238,363	6001
**Sex**		
male	81,143 (65.11%)	1988 (40.87%)
female	38,295 (30.73%)	2744 (56.42%)
not informed	5178 (4.16%)	132 (2.71%)
**Age (years)**		
<1	2956 (2.37%)	243 (5.00%)
01–11	5553 (4.46%)	245 (5.04%)
12–17	2509 (2.01%)	116 (2.38%)
18–44	31,881 (25.58%)	1555 (31.97%)
45–64	34,127 (27.39%)	1037 (21.32%)
>65	30,897 (24.79%)	1169 (24.03%)
not informed	16,693 (13.40%)	499 (10.26%)
**Data entry type**		
Healthcare services	67,899 (54.48%)	3681 (75.68%)
Pharmaceutical companies	41,743 (33.50%)	1140 (23.44%)
Patients/Healthcare professionals	14,795 (11.87%)	42 (0.86%)
Vaccination services	157 (0.13%)	1 (0.02%)
Not informed	22 (0.02%)	0 (0.00%)
**Notified by**		
pharmacist	53,465 (42.90%)	3355 (68.98%)
consumer/non healthcare professional	31,165 (25.01%)	84 (1.73%)
other healthcare professional	29,868 (23.97%)	1211 (24.90%)
physician	7989 (6.41%)	50 (1.03%)
lawyer	157 (0.13%)	2 (0.04%)
not informed	1972 (1.58%)	162 (3.33%)
**Severity criteria**		
other significant events	43,880 (26.62%)	720 (13.26%)
hospitalization	17,514 (10.63%)	278 (5.12%)
death	5033 (3.05%)	12 (0.22%)
threat to life	4526 (2.75%)	239 (4.40%)
persistent incapacity	1950 (1.18%)	65 (1.20%)
congenital anomaly	109 (0.07%)	0 (0.00%)
not informed	91,822 (55.71%)	4115 (75.80%)

For data collection, the categorization of ADRs was based on the SOC, which creates groups by etiology, manifestation site, or purpose. According to these records, the most significant adverse effects associated with opioids included cardiac disorders, skin and subcutaneous tissue disorders, gastrointestinal disorders, and nervous system disorders, all demonstrating high ROR. The ROR, a key metric in pharmacovigilance, measures the disproportionality of ADR notifications by comparing the likelihood of ADR occurrence in exposed versus nonexposed patients. A ROR greater than 1 indicates a positive association between the drug and an increase in ADR notifications, with higher values signifying greater disproportionality.^22–24^ Figure 2 and Supplementary Table 1 present the calculated ROR, with a 95% confidence level [ROR (IC95%)], for the ADRs classified by the main system organ class (SOC) for all opioids and for each drug. The adverse events most related to opioids in general were cardiac disorders [ROR 1.70 (1.50–1.92)], disorders of the cutaneous and subcutaneous tissues [ROR 2.18 (2.05–2.31)], gastrointestinal disorders [ROR 2.88 (2.70–3.06)] and nervous system disorders [ROR 1.19 (1.09–1.31)]. Gastrointestinal disorders were associated with the use of all opioids, with higher ROR for tramadol [ROR 6.94 (6.32–7.63)] and methadone [ROR 4.10 (3.20–5.25)]. In general, fentanyl was the drug in which we observed more associations with ADRs, presenting ROR > 1 in seven of the 11 classifications by SOC, with the greatest ROR for cardiac disorders [ROR 6.23 (5.32–7.30)]. The most common clinical manifestations associated with opioid use were itching (21.48%), constipation (10.96%), nausea (9.75%), and vomiting (7.01%). Erythema was the least reported symptom, appearing in only 2.69% of notifications (Table 2). Additionally, we analyzed the main clinical complications reported in the data set, which included itching (21.48%), constipation (10.96%), and nausea (9.75%). A systematic review encompassing 1,145 patients revealed that ADRs related to opioids are prevalent, with 80% of patients experiencing at least one adverse event.^25^ The most commonly reported manifestations were constipation (41%) and nausea (32%), which align with our findings. Additional ADRs documented included gastrointestinal disturbances, cardiovascular issues, sleep disturbances, endocrine disorders, renal and urinary complications, and long-term effects,^26,27^ many of which were similarly observed in our study.

**Table 2:** Top 10 Principal Clinical Manifestations of Opioid Reports

clinical manifestations	number of notifications
itching	1045 (21.48%)
constipation	533 (10.96%)
nausea	474 (9.75%)
vomit	341 (7.01%)
somnolence	179 (3.68%)
bradycardia	178 (3.66%)
product prescription error	167 (3.43%)
depressed level of consciousness	165 (3.39%)
rash	163 (3.35%)
erythema	131 (2.69%)

Tramadol indicated for the management of mild to moderate pain, and fentanyl, used for epidural analgesia, were the opioids most frequently associated with ADRs. Notably, fentanyl exhibited the strongest association with reports according to SOC classifications. A study analyzed data from 2003 to 2021, resulting in 8769 reports of ADRs associated with opioid use. The findings revealed that tramadol represented the highest proportion of ADR reports at 31.31%, followed by oxycodone at 21.38% and fentanyl at 12.49%.^28^ In a systematic review covering regions of the USA, Canada, Europe, and Australia, Gustafsson et al. highlight that the increasing use of opioids may lead to drug abuse, resulting in tolerance and the need for higher doses to achieve the desired effect. Additionally, the indiscriminate use of opioids without a prescription has contributed to rising abuse rates. The study also reported potential medication administration errors, such as inappropriate or ineffective prescribing practices and both underprescribing and overprescribing and in illegible prescriptions. Medication errors involving opioids can lead to severe adverse effects on SOC classification.^29^

In Brazil, opioid use is primarily limited to the treatment of severe acute pain and chronic oncological pain. There is a lack of published studies that enable effective comparisons; however, opioid prescription practices in Latin America differ significantly from those in high-income countries. For instance, between 2016 and 2018, North America (the United States and Canada) accounted for 60% of global opioid consumption, leading to increased opioid-related harm, including mortality and morbidity, particularly due to the greater availability of prescriptions since the 2000s.^30–32^

The study also aimed to relate genetic factors and population data, recognizing that genetic variations in pharmacogenetics significantly influence individual responses to opioids impacting both therapeutic efficacy and the risk of ADR. Pharmacogenetic information relevant to opioid treatment includes genes involved in pharmacodynamics (*OPRM1, COMT, OPRD1*) and pharmacokinetics (*CYP2D6, ABCB1, SLC22A1, UGT2B7, CYP1B1, CYP3A4, CYP2C19, CYP2B6, SULT1A3*, and *SULT1A1)*. Variants in genes such as *CYP2D6*, *CYP3A4*, and *OPRM1* can alter opioid metabolism, resulting in rapid or slow metabolism of these drugs. This genetic variability may increase the risk of ADRs.^33–36^ When Figures 2 and 3 are considered together, it becomes evident that opioids associated with genes at evidence Levels 1 or 2 (Figure 3) exhibit moderate or large confidence intervals for the ROR (Figure 2) for at least one system organ class. Notably, codeine and methadone demonstrate moderate or large ROR confidence intervals across multiple SOCs. The moderate or large confidence intervals for the ROR indicate variability in the occurrence of adverse effects among the studied samples, which may be attributable to differences in their genetic backgrounds. Therefore, personalized medicine, through genetic testing, presents a promising approach to reducing adverse effects and optimizing opioid treatment.

Figure 3 presents the level of evidence based on the number of studies that found associations between genetic variations and opioid treatment outcomes. *CYP2D6* has Level 1 of evidence for codeine and tramadol and Level 2 for oxycodone. *CYP3A4* and *CYP2B6* have Level 2 evidence for fentanyl and methadone, respectively. In contrast, the other genes are classified as Level 3 of evidence, which is still significant, since several studies reinforce the relevance of genetic variation in these genes and opioid outcomes.

The *CYP2D6* gene was highlighted by 66 studies associating it with tramadol and codeine outcomes, and the other 36 studies with oxycodone outcomes. The *CYP2D6* gene is highly polymorphic, presenting a wide variety of alleles (Supplementary Table 2). This genetic diversity is one of the main determinants of the metabolizing phenotype of individuals and the distribution of types of metabolizers varies according to population. In Supplementary Table 3, we showed the population frequencies of metabolizers. The ultrarapid metabolizers populations range from 0.86 (Asia) to 17.84% (Oceanian) while poor metabolizers range from 0.31 (Oceanian) to 6.5% (Europe). Furthermore, certain drugs share the same genes at evidence Levels 1 and 3. Consequently, when pharmacogenes are analyzed in a patient for a specific treatment, the resulting data may also be applicable to other treatments.

This study highlights significant associations between specific opioids and ADRs, underscoring the critical role of genetic variations in the *CYP2D6* gene, which influences opioid metabolism and ADR susceptibility. These findings highlight the potential of personalized medicine in pain management, enabling safer, more effective treatment strategies tailored to individual genetic profiles. While opioid use in Brazil remains relatively low, further research is essential to better understand and evaluate the adverse effects and therapeutic efficacy of these medications, supporting evidence-based prescribing practices and improved patient care (Figures 1–3).

**Figure 1:**
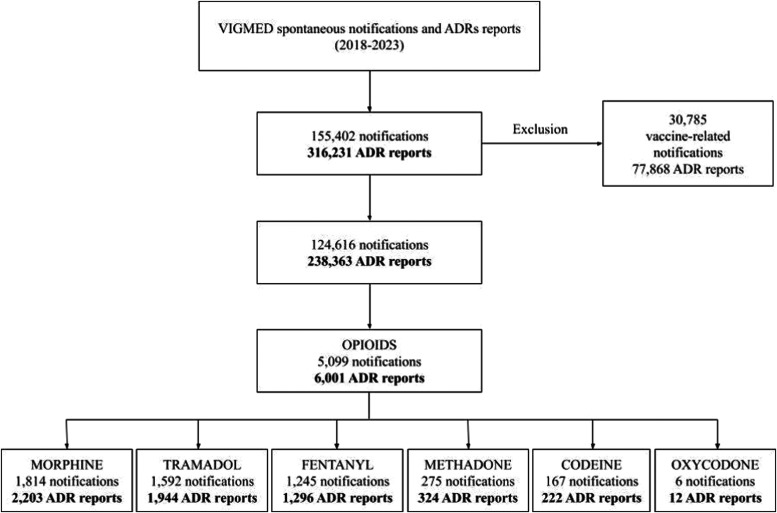
Flowchart illustrating the extraction of data from the VigiMed database, detailing the number of notifications and adverse drug reaction (ADR) reports analyzed, including exclusions of vaccine-related notifications and the breakdown of opioid-related data.

**Figure 2:**
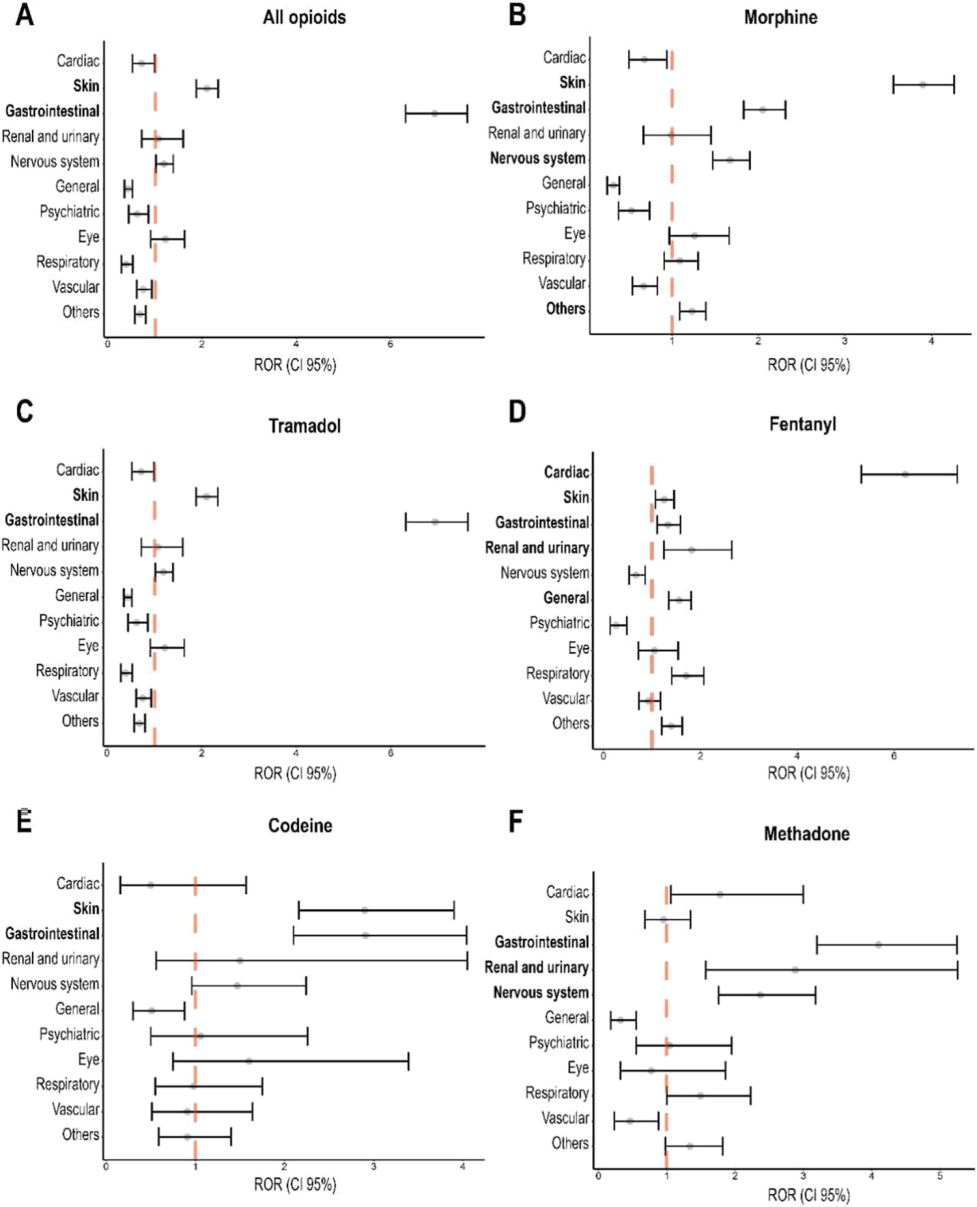
Reporting Odds Ratios (ROR) with 95% confidence intervals for adverse drug reactions (ADRs) associated with opioids, classified by main system organ classes (SOC). Panels show RORs for (A) all opioids, (B) morphine, (C) tramadol, (D) fentanyl, (E) codeine, and (F) methadone.

**Figure 3:**
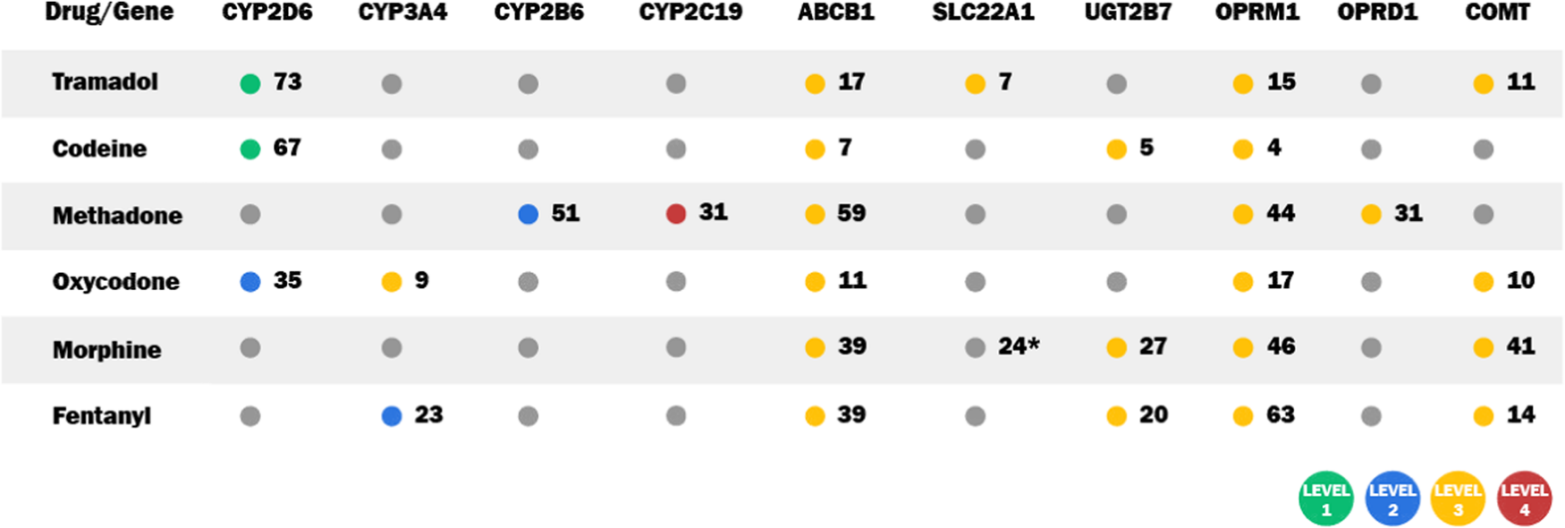
Levels of evidence for the association between genetic variations and opioid treatment outcomes, based on pharmacogenetic studies. The evidence levels from PharmGKB clinical annotations range from 1 (strongest) to 4 (preliminary), highlighting specific genes and their relevance to different opioids. ***CYP2D6*** (Cytochrome P450 2D6), ***CYP3A4*** (Cytochrome P450 3A4), ***CYP2B6*** (Cytochrome P450 2B6), ***CYP2C19*** (Cytochrome P450 2C19), ***ABCB1*** (ATP Binding Cassette Subfamily B Member 1), ***SLC22A1*** (Solute Carrier Family 22 Member 1), ***UGT2B7*** (UDP Glucuronosyltransferase Family 2 Member B7), ***OPRM1*** (Opioid Receptor Mu 1), ***OPRD1*** (Opioid Receptor Delta 1), and ***COMT*** (Catechol-O-Methyltransferase).

## 3 Methods

### 3.1 Data Extraction from VigiMed

This cross-sectional study utilized secondary data from a database of Brazilian spontaneous ADR notifications from VigiMed (Anvisa). The study was based on data analysis regarding routine information from the surveillance of adverse drug reactions from 2018 to April 2023. Figure 1 outlines the data extraction process from VigiMed, including the number of notifications and ADR reports. ADRs were classified using the Medical Dictionary of Regulatory Activities (MedDRA) classification. The reports containing the following drugs classified as opioids were included: tramadol, codeine, methadone, oxycodone, fentanyl, and morphine because these are the most commonly reported opioids associated with adverse drug reactions (ADRs) in Brazil.

We created a database containing (1) general data of ADR groups between Brazilian states, (2) ADR reports by male/female including all system notifications, (3) age; (4) type of entry (healthcare system, pharmaceutical companies, patients, and health professionals or not informed), (5) who (pharmacist, consumers, health professional, physician, lawyer or not informed), (6) ADR coded by MedDRA. In addition, we utilized the System Organ Class (SOC) terminology from the MedDRA classification. This classification system helps to group adverse events by the affected organ or system, providing a standardized framework for analyzing data related to opioid use, including safety concerns and pharmacovigilance.

For statistical analyses, reporting odds ratio (ROR) was used to calculate the strength of the association between opioids and ADRs. The ROR provides the odds of an outcome occurring considering a particular exposure compared with the odds of the outcome occurring without that exposure. When the inferior limit of the ROR’s 95% confidence interval (CI) is greater than 1, the association between ADR and the target drug is considered statistically significant (Rothman et al.,^23^). The ROR measure is defined by the formula [(axd)/(cxb)], as illustrated in Table 3.

**Table 3:** Contingency Table Model of ROR Calculation^a^

	reports with the specific ADR	reports without the specific ADR
reports with the suspected drug	a	b
reports without the suspected drug	c	d

a The ROR can be expressed as ROR = (a/c)/(b/d) = ad/bc. The standard error of ln (ROR) and 95% CI can be calculated by ln (ROR).

### 3.2 Pharmacogenetic Data Sources

The Dutch Pharmacogenetics Working Group (DPWG) and CPIC provide evidence-based clinical guidelines to help practitioners interpret genetic test results and optimize drug therapy.

Pharmacogenetic data of genes associated with opioid safety and response (*CYP2D6*, *ABCB1, SLC22A1, UGT2B7, CYP1B1, CYP3A4, CYP2C19, CYP2B6, SULT1A3, SULT1A1, OPRM1, COMT*, and *OPRD1*) were extracted from PharmGKB (https://www.pharmgkb.org/gene/PA128, https://www.pharmgkb.org/page/cyp2d6RefMaterials and https://www.pharmgkb.org/ampAllelesToTest). This data was used to create Supplementary Tables (frequency of alleles and metabolizing phenotype in the population for the *CYP2D6* gene) and to prepare the figure with the levels of evidence for opioids and pharmacogenes.

The PharmGKB clinical annotations provide detailed information on variant-drug relations (such as ADRs, efficacy, and dose adjustments). These annotations are classified into different levels of evidence that reflect the robustness of the supporting scientific studies that ensure the associations and indicate degrees of confidence. These levels range from **Level 1**, which represents strong evidence, supported by replicated studies and validated guidelines; **Level 2**, which indicates moderate evidence, though studies may not yet be validated across all populations; **Level 3** which suggests an association but with limited replication; and **Level 4** reflects initial hypotheses based on preliminary studies without robust supporting data.^16^

## Supplementary Material







## Data Availability

The data sets generated and/or analyzed during the current study are publicly, available at https://www.gov.br/anvisa/pt-br/acessoainformacao/dadosabertos/informacoes-analiticas/notificacoes-de-farmacovigilancia and https://www.pharmgkb.org/page/cyp2d6RefMaterials.

## Notes

Supplementary Table 1 presents the calculated Reporting Odds Ratios (RORs) with 95% confidence intervals for adverse drug reactions (ADRs) classified by system organ class (SOC), considering all opioids and individual drugs. (PDF)
Supplementary Table 2 summarizes CYP2D6 gene allele frequencies, activity scores, and gene functions across different populations (PDF)
Supplementary Table 3 reports CYP2D6 phenotype frequencies in various populations (PDF)
