# Supplementary material for: Opioids in the Brazilian Healthcare Landscape: Crucial Analysis through Anvisa VigiMed Data and Pharmacogenetic Aspects
Source: ACS Omega. 2025 May 21;10(21):22158–64. doi: 10.1021/acsomega.5c01527 (PMC12138660; doi:10.1021/acsomega.5c01527)
Supplement: Supplementary file 1 [file ao5c01527_si_001.pdf]

Supplementary Table 1: Calculated ROR (IC95%) for the ADRs classified by the main system organ class (SOC) for all opioids and for each drug.

SOC	TOTAL	opioids total	ROR (IC95%)	TRAMADOL	ROR (IC95%)	CODEINE	ROR (IC95%)	MORPHIN	ROR(IC95%)	METHADONE	ROR(IC95%)	FENTANYL	ROR(IC95%)
Cardiac disorders	6,545 (2.75%)	275	**1.70 (1.50-1.92)**	37 (1.98%)	0.72 (0.52-0.99)	3 (1.40%)	0.5 (0.16-1.57)	40 (1.9%)	0.68 (0.50-0.94)	15 (4.79%)	**1.78 (1.06-3.00)**	186 (14.96%)	**6.23 (5.32-7.30)**
Disorders of the cutaneous and subcutaneous tissues	29,357 (12.32%)	1405	**2.18 (2.05-2.31)**	424 (22.75%)	**2.10 (1.88-2.34)**	62 (28.97%)	**2.9 (2.16-3.90)**	746 (35.37%)	**3.9 (3.56-4.26)**	37 (11.82%)	0.95 (0.68-1.35)	186 (14.96%)	**1.25 (1.07-1.46)**
Gastrointestinal disorders	20,478 (8.59%)	1277	**2.88 (2.70-3.06)**	736 (39.48%)	**6.94 (6.32-7.63)**	46 (21.50%)	**2.91 (2.10-4.04)**	341 (16.17%)	**2.05 (1.83-2.31)**	87 (27.80%)	**4.10 (3.20-5.25)**	138 (11.10%)	**1.33 (1.11-1.59)**
Renal and urinary disorders	2,980 (1.25%)	90	1.20 (0.97-1.49)	25 (1.34%)	1.07 (0.72-1.60)	4 (1.87%)	1.5 (0.56-4.05)	26 (1.23%)	0.99 (0.67-1.45)	11 (3.51%)	**2.88 (1.57-5.26)**	28 (2.25%)	**1.82 (1.25-2.65)**
Nervous system disorders	18,903 (7,93%)	560	**1.19 (1.09-1.31)**	173 (9.28%)	**1.19 (1.02-1.39)**	24 (11.21%)	1.47 (0.96-2.23)	265 (12.57%)	**1.67 (1.47-1.90)**	53 (16.93%)	**2.37 (1.76-3.18)**	68 (5.47%)	0.67 (0.53-0.86)
General disorders and conditions at the site of administration	28,611 (12.00%)	430	0.57 (0.51-0.62)	103 (5.53%)	0.43 (0.35-0.52)	14 (6.54%)	0.51 (0.30-0.88)	87 (4.13%)	0.32 (0.25-0.39)	13 (4.15%)	0.32 (0.18-0.55)	218 (17.54%)	**1.56 (1.35-1.81)**
Psychiatric disorders	7,347 (3.08%)	95	0.51 (0.41-0.62)	36 (1,93%)	0.62 (0.44-0.86)	7 (3.27%)	**1.06 (0.50-2.26)**	35 (1.66%)	0.53 (0.38-0.74)	10 (3.19%)	1.04 (0.55-1.95)	10 (0.80%)	0.26 (0.14-0.48)
Ocular disorders	4,941 (2.07%)	137	1.10 (0.93-1.31)	47 (2.52%)	1.22 (0.91-1.63)	7 (3.27%)	**1.60 (0.75-3.39)**	55 (2.61%)	1.26 (0.97-1.66)	5 (1.60%)	0.77 (0.32-1.86)	27 (2.17%)	1.05 (0.72-1.54)
Respiratory, thoracic, and mediastinal disorders	13,671 (5.74%)	317	0.92 (0.82-1.03)	43 (2.31%)	0.39 (0.29-0.53)	12 (5.61%)	0.98 (0.55-1.75)	131 (6.21%)	1.09 (0.91-1.30)	26 (8.31%)	**1.49 (1.0-2.23)**	117 (9.41%)	**1.71 (1.41-2.07)**
Vascular disorders	14.553 (6.11%)	263	0.70 (0.62-0.80)	87 (4.67%)	0.75 (0.61-0.93)	12 (5.61%)	0.91 (0.51-1.64)	88 (4.17%)	0.67 (0.54-0.83)	9 (2.88%)	0.46 (0.23-0.88)	71 (5.71%)	0.93 (0.73-1.18)
Injuries, poisonings, and procedural complications	27,857 (11.69%)	699	1.00 (0.92-1.08)	153 (8.21%)	0.68 (0.57-0.80)	23 (10.75%)	0.91 (0.59-1.40)	295 (13.99%)	**1.23 (1.09-1.39)**	47 (15.02%)	1.34 (0.98-1.82)	194 (15.61%)	**1.40 (1.20-1.63)**

Evento Adverso SOC	N	PERC	N opi	PERC	ROR	LIM INF ROR	LIM SUP ROR
Circunstâncias sociais	821	0,34%	3	0,05%	0,14	0,05	0,45
Distúrbios cardíacos	6.545	2,75%	275	4,58%	1,70	1,50	1,92
Distúrbios congênitos, de família e genéticos	277	0,12%	1	0,02%	0,14	0,02	1,02
Distúrbios do ouvido e do labirinto	1.270	0,53%	11	0,18%	0,34	0,19	0,62
Distúrbios do sistema imunitário	5.199	2,18%	72	1,20%	0,54	0,43	0,69
Distúrbios do sistema nervoso	18.903	7,93%	560	9,33%	1,19	1,09	1,31
Distúrbios do sistema reprodutor e da mama	1.472	0,62%	2	0,03%	0,05	0,01	0,21
Distúrbios dos sistemas hematológico e linfático	6.295	2,64%	2	0,03%	0,01	0,00	0,05
Distúrbios dos tecidos cutâneos e subcutâneos	29.357	12,32%	1405	23,41%	2,18	2,05	2,31
Distúrbios endócrinos	487	0,20%	0	0,00%	0,00	#NUM!	#NUM!
Distúrbios gastrointestinais	20.478	8,59%	1277	21,28%	2,88	2,70	3,06
Distúrbios gerais e quadros clínicos no local de administração	28.611	12,00%	430	7,17%	0,57	0,51	0,62
Distúrbios hepatobiliares	1.631	0,68%	5	0,08%	0,12	0,05	0,29
Distúrbios metabólicos e nutricionais	5.092	2,14%	20	0,33%	0,15	0,10	0,24
Distúrbios musculoesqueléticos e do tecido conjuntivo	9.804	4,11%	101	1,68%	0,40	0,33	0,49
Distúrbios oculares	4.941	2,07%	137	2,28%	1,10	0,93	1,31
Distúrbios psiquiátricos	7.347	3,08%	95	1,58%	0,51	0,41	0,62
Distúrbios renais e urinários	2.980	1,25%	90	1,50%	1,20	0,97	1,49

Distúrbios respiratórios, torácicos e do mediastino	13.671	5,74%	317	5,28%	0,92	0,82	1,03
Distúrbios vasculares	14.553	6,11%	263	4,38%	0,70	0,62	0,80
Infecções e infestações	8.922	3,74%	87	1,45%	0,38	0,31	0,47
Investigações	10.352	4,34%	105	1,75%	0,39	0,32	0,48
Lesões, intoxicações e complicações de procedimentos	27.857	11,69%	699	11,65%	1,00	0,92	1,08
Não Informado	1.134	0,48%	14	0,23%	0,49	0,29	0,83
Neoplasias benignas, malignas e não especificadas (incl. cistos e pólipos)	3.788	1,59%	5	0,08%	0,05	0,02	0,12
Problemas relacionados ao produto	2.731	1,15%	10	0,17%	0,14	0,08	0,27
Procedimentos cirúrgicos e médicos	2.947	1,24%	14	0,23%	0,19	0,11	0,32
Quadros clínicos na gravidez, no puerpério e perinatais	898	0,38%	1	0,02%	0,04	0,01	0,31
	238363		6001				
