# Supplementary material for: Opioids in the Brazilian Healthcare Landscape: Crucial Analysis through Anvisa VigiMed Data and Pharmacogenetic Aspects
Source: ACS Omega. 2025 May 21;10(21):22158–64. doi: 10.1021/acsomega.5c01527 (PMC12138660; doi:10.1021/acsomega.5c01527)
Supplement: Supplementary file 2 [file ao5c01527_si_002.pdf]

Supplementary Table 2: *CYP2D6* gene allele frequencies, activity score, and gene function in different populations.

				POPULATIONS									
**GENE**	**ALLELE**	**ACTIVITY SCORE**	**FUNCTION**	**Brazil***	**African American/ Afro-Caribbean**	**American**	**Central/South Asian**	**East Asian**	**European**	**Latino**	**Near Eastern**	**Oceanian**	**Sub-Saharan African**
CYP2D6	***2**	1.0	Normal function	21.5%	15.45%	21.74%	27.45%	11.92%	18.54%	22.68 %	19.00%	6.11%	17.41%
CYP2D6	***3**	0.0	No function	0.7%	0.33%	0.07%	0.13%	0.01%	1.59%	0.72%	0.43%	0.10%	0.10%
CYP2D6	***4**	0.0	No function	9.4%	4.81%	10.19%	8.96%	0.53%	18.48%	12.05 %	11.41%	1.78%	2.87%
CYP2D6	***5**	0.0	No function	4.6%	5.42%	1.64%	4.18%	4.82%	2.95%	2.92%	1.82%	3.48%	6.21%
CYP2D6	***6**	0.0	No function	-	0.29%	0.25%	0.04%	0.02%	1.12%	0.51%	0.54%	0.00%	0.00%
CYP2D6	***7**	0.0	No function	-	0.02%	0.31%	0.73%	0.01%	0.05%	0.00%	0.29%	0.00%	0.00%
CYP2D6	***8**	0.0	No function	-	0.00%	0.10%	0.00%	0.00%	0.02%	0.00%	0.00%	0.00%	0.00%
CYP2D6	***9**	0.25	Decreased function	1.1%	0.43%	0.71%	0.19%	0.17%	2.75%	1.55%	0.38%	0.00%	0.00%
CYP2D6	***10**	0.25	Decreased function	2.05%	3.82%	1.45%	7.56%	42.84%	1.57%	2.63%	6.77%	5.71%	4.87%
CYP2D6	***12**	0.0	No function	-	0.07%	0.64%	0.00%	0.00%	0.01%	-	0.00%	-	0.21%
CYP2D6	***14**	0.50	Decreased function	-	0.00%	0.00%	0.00%	0.47%	0.00%	0.00%	-	0.00%	0.00%

CYP2D6	***15**	0.0	No function	-	0.00%	0.09%	0.00%	0.01%	0.05%	0.00%	0.00%	0.00%	0.23%
CYP2D6	***17**	0.50	Decreased function	5.6%	16.88%	0.53%	0,05%	0.01%	0.39%	2.33%	3.10%	0.10%	19.36%
CYP2D6	***21**	0.0	No function	-	0.00%	0.00%	0.00%	0.35%	0.00%	-	0.00%	-	0.00%
CYP2D6	***29**	0.50	Decreased function	3.4%	8.74%	0.20%	0.16%	0.01%	0.10%	1.52%	0.78%	0.00%	10.83%
CYP2D6	***31**	0.0	No function	-	0.00%	0.60%	0.00%	0.00%	0.15%	0.75%	0.00%	0.00%	0.00%
CYP2D6	***40**	0.0	No function	-	0.46%	0.00%	0.00%	0.00%	0.07%	-	0.00%	0.00%	1.38%
CYP2D6	***41**	0.25	Decreased function	5.5%	3.71%	2.71%	11.93%	2.32%	9.24%	5.10%	15.36%	3.24%	4.53%
CYP2D6	***42**	0.0	No function	-	0.37%	0.00%	0.08%	0.00%	0.00%	-	0.00%	-	0.10%
CYP2D6	***49**	0.50	Decreased function	-	0.00%	0.00%	0.00%	0.99%	0.00%	-	0.00%	-	0.00%
CYP2D6	***56**	0.0	No function	-	0.16%	0.00%	0.00%	0.00%	0.08%	-	0.00%	-	0.20%
CYP2D6	***59**	0.50	Decreased function	-	0.00%	0.10%	0.00%	0.00%	0.37%	-	0.00%	-	0.00%

*Data extracted from Friedrich et al., 2019 [36] and PharmGKB.
