# Supplementary material for: Opioids in the Brazilian Healthcare Landscape: Crucial Analysis through Anvisa VigiMed Data and Pharmacogenetic Aspects
Source: ACS Omega. 2025 May 21;10(21):22158–64. doi: 10.1021/acsomega.5c01527 (PMC12138660; doi:10.1021/acsomega.5c01527)
Supplement: Supplementary file 3 [file ao5c01527_si_003.pdf]

Supplementary Table 3: *CYP2D6* phenotype frequencies in different populations.

		**POPULATIONS**									
**PHENOTYPE CYP2D6**	**ACTIVITY SCORE**	**Brazil***	**African American/ Afro-Caribbean**	**American**	**Central/South Asian**	**East Asian**	**European**	**Latino**	**Near Eastern**	**Oceanian**	**Sub-Saharan African**
Ultrarapid Metabolizer	≥2.5	3.71%	4.08%	5.14%	1.50%	0.86%	2.33%	4.07%	7.44%	17.84%	3.60%
Normal Metabolizer	1.25-2.25	83.50%	53.79%	64.85%	58.10%	53.83%	49.19%	59.57%	56.54%	63.56%	25.45%
Intermediate Metabolizer	0.25-1.0	7.10%	35.89%	23.07%	28.05%	38.25%	38.25%	29.09%	30.08%	9.55%	33.92%
Poor Metabolizer	0	2.50%	2.35%	2.02%	2.35%	0.79%	6.50%	3.12%	2.20%	0.31%	2.04%
Indeterminate	n/a	3.40%	3.89%	4.92%	9.99%	6.27%	3.73%	4.16%	3.74%	8.73%	35.00%

*Data extracted from Friedrich et al., 2019 [36] and PharmGKB.
